# The mediating role of motivated strategies in the relationship between formative classroom assessment and academic well-being in medical students: a path analysis

**DOI:** 10.1186/s12909-022-03118-y

**Published:** 2022-01-14

**Authors:** Majid Yousefi Afrashteh, Shamsi Rezaei

**Affiliations:** Department of Psychology Faculty of Humanities, University of Zanjan, Zanjan, Iran

**Keywords:** Motivated strategies for learning, Formative assessment, Academic well-being, Medical students

## Abstract

**Background:**

The support of students' academic well-being is one of the main agendas of medical education. For medical students, well-being can help prevent burnout and provides students with grounds for their future healthcare setting. The aim of this study was to examine the mediating role of motivated strategies for learning in the relationship between formative assessment and academic well-being.

**Method:**

The present cross-sectional study was performed on 391 undergraduate students of medical sciences selected by a convenient sampling method**.** The measuring instruments used in this study included motivated strategies for learning questionnaire (Pintrich and De Groot), classroom assessment approaches questionnaire (Yousefi Afrashteh et al.) and Academic well-being Questionnaire (Pietarinen et al.). In order to analyze the data, SPSS-26 software was used for descriptive statistics and correlation matrix, and LISREL-10.20 software was used to do path analysis and determine the relationships between variables within the model.

**Results:**

Findings showed that formative assessment is a significant resource in shaping subscale of motivated strategies for learning (self-efficacy, intrinsic value, test anxiety, cognitive strategies and self-regulation). Moreover, the results demonstrated that the self-regulated learning strategies is a crucial determinant of academic well-being and is a mediator between formative assessment and academic well-being.

**Conclusion:**

These findings suggest the important value and necessity of formative assessment in medical science classes which can indirectly lead to improve students’ academic well-being.

## Introduction

Studies show the prevalence of psychological problems such as depression and anxiety and psychological distress in medical students in comparison to general population, increasing with the years of training [1]. Psychological well-being of medical students is a growing concern in medical education in Iran [2–4]. Understanding the role of educators and programs in supporting their psychological well-being is an important issue [5]. Psychological well-being, as research has shown, encompasses all aspects of human performance and affects academic outcomes, meaning active participation in work and leisure, experiencing positive emotions, developing a sense of autonomy, and promoting the purpose of life [6, 7]. One of the concepts in the framework of positive psychology in the field of education is academic well-being, which is considered as students' attitude to education and has components such as homework skills, satisfaction in academic performance and academic engagement [8]. Students who are emotionally and cognitively involved in learning spend more time and effort studying and adapt to their academic needs appropriately. Learners' academic well-being is considered as an important and comprehensive indicator in emotional and cognitive involvement in the educational process [9].

One of the factors that seems to be related to the attitudes toward lesson and academic well-being is the formative assessment [10], the goal of which is to improve the learning process, and for that reason is called assessment for learning as well. The formative assessment is defined by Assessment Reform Group [11] as a process of seeking and interpreting evidence by the learners and their teachers in order to decide regarding where the learners are located in the learning of their tasks, where they need to go, and how to best get there. According to Moss and Brookhart [12], the formative assessment can be defined as a process in which teachers and students provide feedback during the instruction process in order to improve the learning and teaching activities, with the purpose of increasing the extend of student achievement. The formative assessment has four main components included (i) the expression of learning goals and success criteria, (ii) the improvement of the quality of inquiry/dialogue, (iii) the fairness and improvement of the quality of marking/feedback, and (iv) sing the usage of self and peer evaluation [10, 13, 14]. Barana and Marchisio [15] believe that formative assessment, along with increasing scores, increases engagement and motivation in learners. This assessment is done during the training period when the teacher's educational activity is ongoing [16]. Due to the mission of medical education in the country's health system and the need for valid assessments of the competencies expected of medical science graduates, it is necessary to pay more attention to classroom assessment. In formative assessment, guidance evaluation and feedback are of particular importance [17].

Motivated strategies for learning are one of the variables related to both formative assessment and academic well-being. So it seems to play a mediating role [10]. Self-regulated Learning Strategies include self-efficacy, internal value, test anxiety, cognitive strategies, and self-regulation [18]. Motivated strategies for learning are a set of personal and social criteria that people refer to before performing or avoiding an action. These motivational criteria are formed following the approval or disapproval of behavior by important people in life [19]. Self-efficacy is defined as "a person's judgment of how he or she can handle a situation well (or badly) given the skills he or she has and the situations he or she faces."[20]. Self-efficacy beliefs affect the quality of human functioning through cognitive, motivational, emotional and decision-making processes. These beliefs shape the expectations of the outcome, the causal attributions of successes and failures, and the ways in which individuals motivate themselves and endure adversity. In addition, self-efficacy affects individuals' beliefs in coping with abilities, emotion regulation mechanisms, and vulnerability to stress and depression [21]. In internal value, the student believes that the results of progress are due to personal effort rather than external factors such as luck or the teacher [22]. Test anxiety is an unpleasant emotional state that has special behavioral and psychological consequences and is experienced in formal exams or other evaluation situations [23]. Cognitive strategies are strategies that increase the student's ability to process information more deeply, transfer and apply information in new situations, and enhance and maintain learning [24]. Self-regulation refers to the process of managing oneself to achieve a long-term goal. In self-regulated learning, learners fall into a recursive (not necessarily linear) triad: (1) planning stage—goal setting and task analysis; (2) performance stage—monitoring behaviors, emotions and motivation; (3) evaluation stage—self-reflection based on feedback. Thus, self-regulation is an ongoing and participatory process that includes: planning, implementation, monitoring and metacognitive evaluation of the individual's efforts for the goal [25].

Many studies have examined the relationship between formative assessment and other factors. For example, in an experimental study by Rakoczy et al. [26], it was shown that in formative assessment conditions, feedback was more useful and self-efficacy was higher, and interest was increasing. Yousefi Afrashteh's findings [27] showed that formative assessment in medical science classes leads to improve students' academic motivation and greater use of metacognitive strategies in learning. The relationship between self-efficacy and academic well-being has also been investigated. For example, the study of Salmela-Aro and Upadyaya [28] showed that academic self-efficacy has a positive relationship with academic engagement (one of the components of academic well-being). The research of Sahraei et al. [29] also showed that part of the distribution of academic well-being can be explained by academic self-efficacy. Among the modeling studies that have been conducted in recent years in the field of identifying mediating variables in formative assessment, we can mention the research conducted by Leenknecht et al. [30]. The results showed that more perceived use of formative assessment is associated with more feelings of autonomy and competence, and more autonomous motivation.

**Fig. 1:**
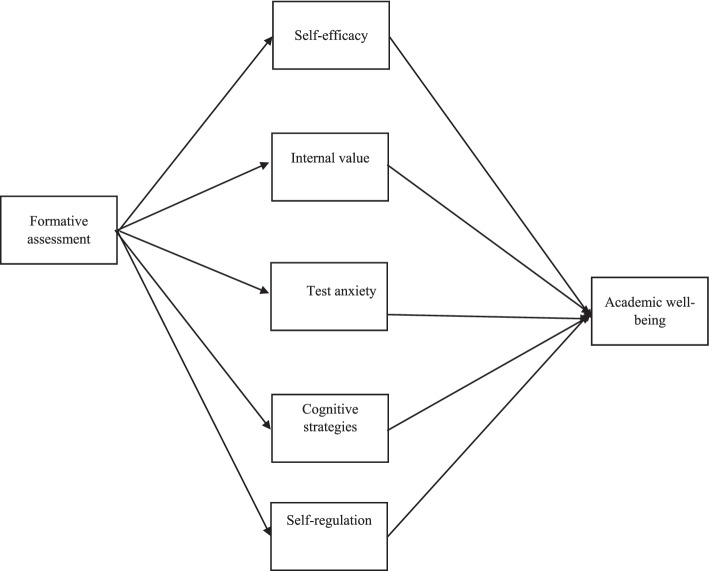
Conceptual model of research

Students' psychological well-being is an area in medical education that has become more important in recent years due to the increasing prevalence of anxiety among medical students [31]. Students' learning environment plays an important role in their psychological well-being [5]. In particular, the effectiveness and quality of assessment and evaluation is a key factor in promoting a positive learning environment [17]. As a result, in this study, we have focused on academic well-being, which is one of the important indicators in evaluating educational systems [32]. Findings indicate the importance of formative assessment factor on psychological variables such as well-being, self-efficacy and motivation [26, 30, 32] as well as the relationship between motivated strategies for learning and academic well-being [29]. However, similar studies have not addressed the mediating role of motivated strategies for learning as an influential factor. Therefore, our goal is to supplement previous research and explain the causal relationships between formative assessment, motivated strategies, and academic well-being. Therefore, considering the necessity of studying the function of formative assessment in students' psychological well-being and considering the gaps in the research background in this field, the main purpose of this study is to identify the relationship between formative assessment and psychological well-being according to the role of motivational beliefs. The theoretical model is shown in Fig. 1.

## Methods

### Design and data collection

The present cross-sectional study was performed on 391 undergraduate students of medical sciences in the 2021 selected by a convenient sampling method. Due to the prevalence of COVID-19 pandemic and the need to maintain social distance to collect data, the questionnaires were first designed on the web and sent to students. On the first page, a questionnaire about study objectives and entry criteria, and how to respond was mentioned. Students were asked to first read the consent form and entry criteria and sign the form if they agreed. They then completed the demographic information on the second sheet. Finally, they answered three questionnaires the Motivated Strategies for Learning Questionnaire (MSLQ), classroom assessment approaches questionnaire (CAAQ) and Pietarinen academic well-being.

### Measures

Motivated Strategies for Learning Questionnaire (MSLQ) was developed in 1990 by Pintrich and De Groot [18]. This questionnaire with 44 items is organized in two parts motivational beliefs (22 items) and self-regulated learning strategies (22 item). Motivational beliefs include the three subscales of self-efficacy, intrinsic value and test anxiety and self-regulated learning strategies include the two subscales of cognitive strategies use and self-regulation. Responses were scored on a 7-point Likert scale [1–7]. Pintrich and De Groot reported the reliability of the five subscales of self-efficacy, intrinsic value, test anxiety, cognitive strategies use and self-regulation 0.89, 0.87, 0.75, 0.83 and 0.74, respectively. The reliability of MSLQ in the present study with Cronbach's alpha for the five subscales was 0.79, 0.80, 0.82, 0.78 and 0.77, respectively.

The classroom assessment approaches questionnaire (CAAQ) was used to assess students' perceptions of the teacher's classroom assessment. This questionnaire with questions such as “Did you receive any feedback on how you learned while studying?" was designed by Yousefi Afrashteh, Siami and Rezaei [32] in Iran and was used to identify the teacher's classroom assessment approach. In this questionnaire, the higher the score obtained, the more the teacher uses the formative assessment. The reliability coefficient of this instrument was 0.72 in Yousefi and Siami research. The reliability of this questionnaire to Cronbach's alpha index in Iranian medical students was 0.78 [33]. The reliability of this questionnaire in the present study was 0.78.

The questionnaire of Pietarinen et al. [34] was used to assess academic well-being. This questionnaire has 11 questions and with a five-point Likert scale (from strongly disagree to strongly agree) with questions such as "Going to university does not seem necessary." which measures academic well-being. The high score represents more academic well-being. The reliability of this questionnaire was 0.70 in Mohanna and TalePasand research [35] and 0.79 in Ghadmapour et al. research [36]. The reliability of the present study was calculated using Cronbach's alpha coefficient equal to 0.72.

### Statistical analysis

In order to analyze data, SPSS-26 software was used for descriptive statistics and correlation matrix, and LISREL-10.20 software was used to do path analysis and determine the relationships between variables within the model. Path analysis is a method by which the relationship between several independent variables and several dependent variables is evaluated simultaneously. The advantage of this method over multiple regression, in addition to the possibility of examining more than one dependent variable simultaneously, is the study of three direct, indirect and total effects. In the present study, in addition to several direct effects, several indirect effects are considered. Indirect effects are related to the effect of Formative assessment on academic well-being through mediating variables. The mediating variables in this study, as shown in Fig. 1, are: s elf-efficacy, internal valuation and test anxiety.

The overall model fitness is evaluated by several goodness of fit indices to assess the extent to which the data supports the conceptual model. Various goodness of fit indices used in this study include the likelihood ration chi-square (χ2), the ratio of χ^2^ to degrees of freedom (χ2 /df), the goodness of fit index (GFI), the adjusted goodness of fit (AGFI), the root mean square error of approximation (RMSEA) and goodness fit Comparative Fit Index (CFI) [37].

## Results

Table 1 reports the socio-demographic information of the participants. Out of 391 students participating in this study, 23% of the participants were in the age group of less than 20 years and 47% in the age group of 20–25 years. 25% of the participants were married. 72% of the participants were undergraduate students, 21% were postgraduate students and 7% were PhD students. 51% of them were employed while studying. 22% of the participants studied in the School of Health, 28% in the School of Paramedical Sciences, 23% in the School of Rehabilitation and 27% in the School of Nursing and Midwifery. More details are reported in Table 1.

**Table 1:** Demographic statistics of the participants

Characteristic	Frequencies	Percent
**Age**		
< 20	92	23
20–25	185	47
25–30	70	18
30 <	44	2
**Marital status**		
Married	98	25
Single	293	75
**Grade**		
Undergraduate	282	72
Masters	82	21
PhD	27	7
**Simultaneous job**		
yes	200	51
no	191	49
**School**		
Health	85	22
Paramedical	111	28
Rehabilitation	89	23
Nursing and midwifery	106	27

Table 2 shows the descriptive information including mean and standard deviation for research variables. In addition, Pearson correlation is reported to determine the relationship of all variables included in the path model. The mean and standard deviation of Academic well-being are 40.40 and 8.27, respectively. The correlation coefficient of academic well-being with formative assessment was 0.02, with self-efficacy was 0.39, with internal value was 0.28, with test anxiety was 0.26, with cognitive strategies was 0.19 and with self-regulation was 0.28. Apart from the relationship between academic well-being and formative assessment, other correlation coefficients are significant at the level of 0.001.

**Table 2:** Descriptive statistics for research variables and correlation coefficient between them

variable	M	SD	Correlation matrix					
			1	2	3	4	5	6
1-Formative assessment	29.89	5.38	-					
2-Self-efficacy	36.54	5.90	0.36^***^	-				
3-Internal value	29.98	5.21	0.25^***^	0.25^***^	-			
4-Test anxiety	14.90	3.14	-0.28^***^	-0.30^***^	-0.39^***^	-		
5-Cognitive strategies	55.13	7.79	0.30^***^	0.27^***^	0.16^**^	0.30^***^	-	
6-Self-regulation	41.74	6.36	0.24^**^	0.26^***^	0.29^***^	0.27^***^	0.21^***^	-
7-Academic well-being	40.40	8.27	0.02	0.39^***^	0.28^***^	-0.26^***^	0.19^***^	0.28^***^

*M* Mean, *SD* Standard deviation

^**^P < 0.01; *** *P* < 0.001

The results of path analysis to investigate direct, indirect and total relationships are reported in Table 3.

**Table 3:** Path coefficients for direct, indirect and total relationships

Dependent	Predictors	Standard estimate	*t*-value	*P* value
Self-efficacy	Formative assessment	0.24	4.95	0.001
Internal value	Formative assessment	0.17	3.52	0.001
Test anxiety	Formative assessment	-0.20	-4.01	0.001
Cognitive strategies	Formative assessment	0.19	3.52	0.001
Self-regulation	Formative assessment	0.16	3.11	0.001
Academic well-being (R2 = 0.50)	Self-efficacy	0.26	5.21	0.001
	Internal value	0.21	4.23	0.001
	Test anxiety	-0.20	-4.09	0.001
	Cognitive strategies	0.16	3.33	0.001
	Self-regulation	0.22	4.32	0.001
Indirect effect of formative assessment to Academic well-being		0.16	4.08	0.001
Total effect of formative assessment Academic well-being		0.16	4.08	0.001

**Fig. 2:**
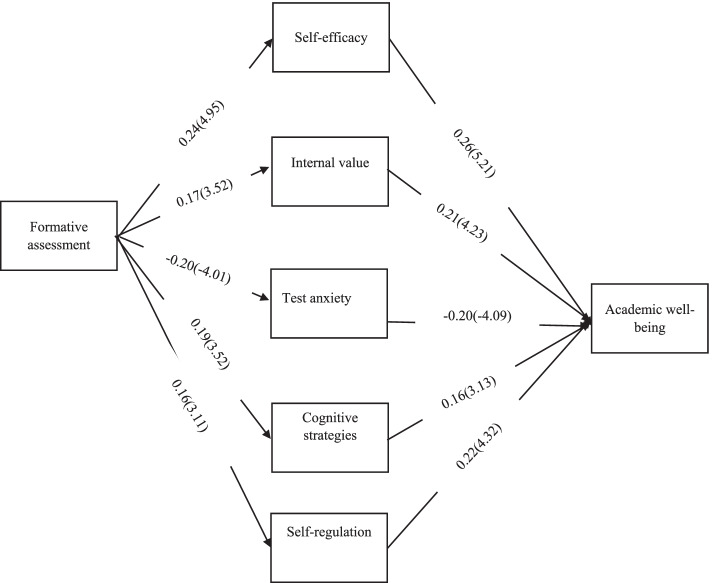
standard estimate (and t-value) for relationship between variables

Standard estimate (and t-value) for relationship between variables has showed in Fig. 2

The goodness-of-fit indices reported in Table 4 shows that the analyzed model has an excellent fit (*P*-value = 0.001; chi square = 7.05; df = 3; chi square/df = 2.35; RMSEA = 0.06; GFI = 0.97; AGFI = 0.95).

**Table 4:** The goodness of Fit Indices for the Models

Index:	CFI	AGFI	RMSEA	χ2	df	χ2/df	*P*-value
Value:	0.97	0.95	0.06	7.05	3	2.35	0.001

*CFI* Comparative Fit Index, *AGFI* Adjusted Goodness Fit Index, *RMSEA* Root Mean Square Error of Approximation, χ2/df: χ2 to the degree of freedom index

Goodness-fit indices are reported in Table 4.

## Discussion

Considering the importance of psychological well-being in the field of education, in the present study, an attempt has been made to study its relationship with motivated strategies and formative assessment. In addition, the influence of these factors on each other was examined. The final research model indicates a good fit with the research data. To examine the indirect relationship between formative assessment and academic well-being, it is necessary to confirm the relationship between formative assessment and motivational beliefs and self-regulated learning strategies with academic well-being. The relationships of these variables were examined and all of them were significant. These findings are consistent with the results of Rakoczy et al. [25], Naseri, and Karshki [38].

Reviewing the path coefficients in the model indicates that all path coefficients except the path coefficient of formative assessment to academic well-being (β = 0.03) are significant at the level of 0.05. Another finding showed that formative assessment has the ability to predict motivational beliefs and self-regulated learning strategies. The values of direct effects of the model indicated that the highest correlation between formative measurement and the variable of motivational beliefs was related to the self-efficacy dimension and the lowest correlation was related to the self-regulatory dimension. These findings are consistent with the findings of Granberg, Palm and Palmberg [39] and Hameed and Akhter [40]. A review of theoretical and empirical foundations shows that the direct effect of formative assessment on self-efficacy can be explained by emphasizing the role of performance expectation. Byars-Winston et al. [41] believe that self-efficacy beliefs are theoretically created and maintained by four classes of experiences that Bandura refers to as sources: performance success, learning succession, verbal persuasion, and emotional arousal. In the meantime, the success of a performance or personal experience has by far the strongest unique connection with self-efficacy beliefs. On the other hand, studies have shown that formative assessment has a positive effect on learning and can improve the teaching and learning process by providing effective feedback [17]. In fact, it can be inferred from these two studies that formative assessment provides a good basis for improving learning processes and ultimately academic achievement. Thus, recalling past efforts for well-judged academic assignments increases self-efficacy. In other words, a person's personal history of having the ability to study provides primary information about self-efficacy in the present. Also in the present study, internal value and test anxiety were also affected by formative assessment. Explaining this finding, it can be said that formative assessment facilitates active participation in the learning environment and allows students to receive effective feedback. Reeve believes that performance feedback in its various forms—from homework, from self or others—is emotionally important and activates the student. He may feel so satisfied and competent that he sets higher and more difficult goals for himself. In this case, by accepting the goal of mastery, the person may overcome the challenges according to the criteria set by him and experience more inner motivation [19]. The results of Sanaeifar and Nafarzade have also shown that formative assessment has a significant effect on reducing test anxiety [42]. One possible reason for this finding could be that when learners are exposed to formative assessment, their self-confidence increases and they gradually improve their sense of test and reduce their level of anxiety. Also, the results of the present study showed that cognitive and self-regulatory strategies were influenced by formative assessment. This finding is consistent with various studies [43, 44]. Explaining this result, it can be said that formative assessment improves the mental activities used to store and maintain information by stimulating learners' attention and concentration. [26]. Students' use of cognitive strategies increases their awareness of the strategies, which results in strengthening metacognition and self-regulation [45]. Formative assessment also provides for learner with the opportunity to learn about learning and its strengths or weaknesses. By receiving feedback and information, the student evaluates and reflects on them, which in turn leads to a more conscious pre-performance foresight. As noted in the review of the research background, self-regulation is a cyclical process that includes planning, performance, and evaluation.

Another result of this study shows that motivated strategies affect academic well-being. Academic well-being has a positive relationship with self-efficacy, internal value, cognitive and self-regulatory strategies and a negative relationship with test anxiety. Consistent with the findings of the present study, the results of sahraei, shokri, khanbani and hakimi showed that self-efficacy have the ability to predict academic well-being [28]. Lee and Jeon also showed that self-efficacy had the greatest effect on the burnout component of medical student’s well-being [46]. Bandura states that after self-efficacy beliefs are formed, they affect human performance in different ways. Learning is always accompanied by problems and failures to some extent. Self-efficacy after such obstacles leads to a rapid improvement in self-belief and increases effort and endurance. People with strong self-efficacy pay attention to the necessities of the task and show enthusiasm, interest and optimism. In contrast, people with poor self-efficacy pay attention to personal shortcomings and suffer from pessimism, anxiety, and depression. Bandura states that the main cause of anxiety is low self-efficacy [47, 48]. These findings are important hints for adaptation and well-being in education. Motivational beliefs are related to the way we think, feel and behave positively and productively. Students with poor self-efficacy feel that they make little progress, and this feeling leads to dissatisfaction and negative emotions. Such emotional and motivational states, if experienced too much, will ultimately erode academic well-being.

The proposed model also showed that formative assessment is indirectly related to academic well-being through the mediation of motivational beliefs (self-efficacy, internal value, test anxiety and cognitive and self-regulation strategies). According to this model, it can be said that formative assessment promotes students' motivational beliefs and promotes their beliefs of self-efficacy, internal value, cognitive and self-regulatory strategies, and academic well-being. In this case, a study that examined the relationship between formative assessment and academic well-being according to the proposed model of the present study was not found, so the results of this study are compared with the research of Leenknecht et al. [29]. In their model, formative assessment and learning motivation are related, and the basic psychological needs of students mediate this relationship. The results of their study showed that the perception of formative assessment was associated with a greater sense of autonomy and competence as well as greater motivation to learn. Also, the findings of the present study are consistent with the findings of Rakoczy et al. [25] and Yousefi Afrashteh [26]. Motivational beliefs are influenced by formative assessment [38] and also academic well-being is influenced by motivational beliefs [28]. Formative assessment facilitates active participation in the learning environment and enables students to receive immediate feedback [49]. Receiving feedback allows students to be aware of their own progress toward the goal. Formative assessment provides a positive reinforcement for students to learn small units of the lesson. According to Tolman and Gleitman reinforcers are not necessary for learning but are important for motivating [50]. On the other hand, enhanced motivational beliefs increase academic well-being by improving academic performance and reducing academic stress [28, 51]. Formative assessment helps students monitor their progress. This makes cognitive learning strategies more effective and improves self-regulation, which in turn promotes their learning and creates the belief that learning is accessible and controllable, which in turn It leads to higher self-efficacy, internal value, and lower test stress and anxiety, and may ultimately lead to increased academic well-being.

Although the present study provides information in the context of motivational beliefs, academic well-being and formative assessment, the research sample (a group of medical students) raises limitations in the field of generalized findings that should be considered. Therefore, it is suggested that similar studies be conducted in other universities. Also, considering the use of path analysis in the present study, the conclusion of cause and effect should be made with caution. The aim of this study was to test several causal hypotheses, but the results cannot achieve the direction of causality. It is suggested that future research provide better explanatory and experimental conditions through more controlled studies. Based on the results, specific applications can be recommended for medical education planners. Considering the relationship between formative assessment and students' motivated strategies and academic well-being, it is suggested that the medical education system pay special attention to classroom assessment methods and encourage teachers to use the formative assessment approach.

## Conclusion

In the proposed model of this research, formative assessment is the underlying factor that explains academic well-being through the mediation of motivational beliefs. The importance of this finding is that formative assessment can improve students' academic well-being due to its effect on motivational beliefs. Accordingly, it is suggested that research in this field be continued by identifying the optimal educational methods based on formative assessment and individual factors of learners.

## Data Availability

The datasets during and/or analyzed during the current study available from the corresponding author on reasonable request.

## Abbreviation

COVID-19: Coronavirus disease 2019
